# GU Evaluation and Management of Renal Transplant Candidates and Recipients

**DOI:** 10.1100/tsw.2004.43

**Published:** 2004-06-07

**Authors:** Peter N. Bretan

**Affiliations:** Renal Transplant Service, The University of California at San Francisco Medical Center, San Francisco, CA, USA

## Abstract

There are more than 200,000 end stage renal disease (ESRD) patients who are potential transplant candidates and more than 100,000 previously transplanted renal recipients with functioning allografts in the United States . Fifty-seven percent of these patients are male and forty percent are greater than 50 years of age . Diabetes is the most common cause of kidney failure. It is evident that many patients are at high risk for development of urologic problems and thus it is estimated that the average urologist will care for up to ten of these patients yearly. Thus a review of the genitourinary (GU) evaluation and management of these patients is timely.

## PERITRANSPLANT MALIGNANCY

Which patients will benefit from pretransplant renal cancer screening? What constitutes a safe waiting time prior to transplantation after removal of a cancer? These questions are common and will be now overviewed. Chronic renal failure is a risk factor for development of renal cell carcinoma and a pre malignant state known as acquired renal cystic disease. Because of this, baseline ultrasonography is used to rule out solid native renal masses pre transplantation. In addition, other urologic questions can be answered, such as the presence of hydroureteronephrosis or calculi disease (which is rare). Any solid masses should be considered malignant. Questionable renal masses should be followed. Full urologic evaluation should be carried out in those patients with flank pain, history of renal mass or gross hematuria. An absolute contraindication of renal transplantation is presence of malignancies. A safe waiting time prior to transplantation after removal of tumors depends on their grade and stage. The range is anywhere from two years for low metastatic potential tumors, to six years for patients with high grade and stage tumors. The majority of post transplant tumor recurrences occur within two years [5]. Because many of the patients are males over the age of 50, the presence of a prostatic nodule or elevated prostatic specific antigen (PSA) should be evaluated with a sexton prostate needle biopsy prior to transplantation. Nevertheless, even with the presence of localized cancer, the risk of metastatic disease should be thoroughly considered prior to transplantation.

## WHO NEEDS A PRE TRANSPLANT GU WORK-UP?

Routine use of voiding cystourethrograms (VCUG) usually does not yield any abnormalities that require surgical corrections [6]. This test should be reserved for those patients with urologic anomalies associated with the cause of their renal disease. For patients with significant post void residuals (PVR), further evaluation may be required to rule out bladder outlet obstruction. Small bladder capacities of less than 100 cc may indicate spastic bladders, which are reported to be associated with a reduced two year allograft survival of 55% compared with 92% for larger bladders, and these differences were unrelated to immunologic (rejection) losses [7].

Renal failure associated urolithiasis and nephrocalcinosis can be characteristic of primary hyperoxaluria (PH-1). This is a rare autosomal recessive inborn error of metabolism consisting of loss of a liver specific perisomal enzyme alanine glyoxylate amino transferase, causing loss of glyoxylate amination to glycine. Thus glycosylate accumulates which is subsequently oxidized to oxalate. Oxalate excretion by the kidney leads to urinary precipitation of calcium oxalate. The majority of these patients (greater than 90%) develop renal failure before the age of 20. Thus, adolescent patients presenting with bilateral urolithiasis and ESRD should be screened for PH-1 with a 24 hour urine collection for urinary oxalate. However, most of these patients will have decreased urine production and a serum oxalate and liver biopsy for amino transferase assay may be the only way to diagnose PH-1 (in oliguric renal failure patients). Without proper diagnosis, the risk of rapid precipitation of serum oxalate will adversely affect the renal allograft [8].

### PRE Transplant Bilateral Native Kidney Nephrectomies

Native kidney nephrectomies are seldom required prior to transplantation. Nevertheless the indications are: pyelonephritis, renin mediated hypertension, malignant disease, and unresolving severe nephrotic syndrome. Large polycystic kidneys can be removed prior to transplantation. The patient should be informed that transfusions may be required and that they may stimulate antibody production which can sensitize the patient so that subsequent cross matching may be incompatible with the possibility of transplantation. Small kidneys can be removed via dorsal or laparoscopic approaches [9].

### Surgery For Vesicoureteral Reflux

Neurogenic bladders causing severe reflux may require augmentation. If the native ureters have not been reimplanted, both native dilated ureters can serve as vascularized patch grafts for augmentation cystoplasty. There is an absolute requirement that a low pressure bladder be present prior to transplantation. Concerns for isolated vesicoureteral reflux per se (without high bladder pressures) has been challenged recently and there is growing sediment that reflux by itself may not predispose to urinary tract infections [10].

### Bladder Outlet Obstruction and Timing of Therapy

Incomplete urethral strictures or benign prostatic hypertrophy can be surgically corrected post transplantation. Pre transplant correction should only be undertaken if adequate ultrafiltration fluid production exist which can prevent the high incidence of bladder neck contractures and subsequent stenosis seen in renal failure oliguric patients. The occurrence of a defunctionalized bladder because of prolonged anuric (greater than 2 years) is a risk factor for post transplant urinary retention, however, this can not be differentiated from true bladder outlet obstruction and a post transplant adaptation period (up to 2 months) may be required for the bladder to expand via continuous urine flow. During this period, the defunctionalized bladder can safely adapt via ìcyclingî with a suprapubic tube or intermittent self catheterization. The former is preferred because of ease of use, and the ability to check for frequent post void residuals. In addition, high pressure can be completely prevented thus minimizing the occurrences of urinary tract infection and possible uretero vesicle anastomotic leaks. If urinary retention persists greater than two months after transplantation and medical therapy (ie, Hytrin) has been exhausted, a transurethral resection of the prostate should be considered. The use of other less invasive procedures (ie catheter microwave ablation) should be thoroughly discussed with the patient knowing the risks, complications, and alternatives.

## Pre-transplant Reconstruction

When the bladder is not reparable or usable with acceptable results, ileal conduit, continent urinary diversions, and/or bladder augmentation may be required [11-13]. Each method has an inherent specific complication profile and should be thoroughly reviewed with individual patients prior to use. The use of complex procedures such as gastrocystoplasty has been questioned lately [14-17]. As previously mentioned, the native dilated ureter can be used to augment the bladder and it is the procedure choice in neurogenic bladder associated with severe reflux [18].

Established native kidney cutaneous ureterostomies can be used for transplant drainage by performing ipsilateral transplant uretero native ureterostomy. This procedure should be considered as a preferred alternative to ileal conduit use. Special selection of these patients include those with no history of stomal stenosis, recurrent urinary tract infections, or pyelonephritis. Two year follow-ups in a limited number of patients have yielded excellent results [19].

## Defunctionalized Bladder

Those with a chronic history of anuria prior to transplantation are at significant risk for development of defunctionalized bladders especially if the patients are male, with anuria of greater than two - three years duration. Treatment with post transplant intermittent self catheterization (for patients with enough dexterity to adequately perform this) or placement of suprapubic tube at the time of transplantation are associated with diminished urinary leakage and UTIs. Chronic Foley placement post transplantation are associated with significant urinary tract infections and does not permit the bladder to ìcycleî and develop into a low pressure reservoir during post transplant period of adaptation.

## Delayed Ureteral Anastomotic Stenosis

Distal ureteral obstruction can occur anywhere from 0.5% - 9% [20, 21] and reestablishing patencies in these cases can be difficult. The use of a percutaneous nephrostomy tube as a temporizing measure followed by antegrade balloon dilation have reported durable success rates of 40% - 60%. The open procedure is often associated with significant morbidity and bleeding. More recently, the endopyelotomy Acucise ureteral cutting balloon has led to satisfactory outcome after failure of dilation [22]. This procedure is associated with minimal morbidity , thus should be considered prior to any open procedures.

## Vesicoureteral Reflux

As previously reviewed, vesicoureteral reflux (VUR) pre or post transplant and its significance has been controversial. Multiple studies have shown there is no significant correlation between the degree or presence of VUR with: subsequent graft function, hypertension, rejection, or the number of urinary tract infections [23-26]. When VUR exists, a thorough urologic exam should be performed to rule out bladder outlet obstruction, spastic bladder, or a defunctionalized bladder.

## Urolithiasis

The risk of urolithiasis after renal transplantation (URT) is not different for renal transplant recipients (0.3%) compared to that of patients in the general non transplant population (0.1%). However, bladder drained pancreas/renal transplant patients seemed to have significantly greater risk (1.7% incidence) for URT ($p < 0.01$) [27-29]. Most of these are bladder stones. Regardless, with timely and proper treatment, graft damage and loss can be minimized with excellent long term subsequent function.

## CONCLUSION

Transplant patients will continue to grow in number thus requiring greater involvement of the general urologist. Urologic aspects in these patients are more similar than different compared to the general non transplant population, however, urologic issues are much more crucial in the setting of immunosuppression and as a consequence of the demographics of these high risk patients. This overview has attempted to answer many of the questions found in regard to peritransplant urologic evaluation and treatment of these patients.
